# Recent Progress in Genito-Urinary Surgery

**Published:** 1914-03-07

**Authors:** 


### MODERN METHODS.

### Recent Progress in Genito-Urinary Surgery.

The last ten or twelve years have witnessed progress in the art, and even more in the science, of the surgery of the urinary system which will compare favourably with that achieved in any branch of therapeutics. So much has this been the case, so rapid and revolutionary have been the changes, that there are many practising and operating surgeons who do not realise how vast they have been. At the beginning of this century, when Sir Henry Morris' text-book was the standard authority on surgical diseases of the kidney, general surgeons regarded themselves as fully competent to deal with all genito-urinary diseases which required surgical treatment; and they certainly did at that day bring to bear on their cases knowledge and experience as complete as that possessed by anyone else. Since then, however, a band of active surgeons has devoted a decade of unremitting labours to the exclusive study of this branch of surgery, and conditions are vastly changed as a consequence.

Unfortunately, the lapse of time has been so short that there are still great numbers of surgeons in active practice who have not realised the degree of progress that has been made. They still consider themselves perfectly competent to deal with stone, tuberculosis, cancer, and the other diseases of the urinary tract which may require operation, for the reason that they do not comprehend the extent to which the march of science has left them behind. We say advisedly that there are many hospital surgeons in London to-day (and the same is true of the provinces), men of high reputation and established name, who have no comprehension of their own state of ignorance in respect of urinary surgery, and are failing, in consequence, to afford some of their hospital patients the best skill and attention such as the latter have commanded in the past. There are many surgeons holding appointments at our large hospitals who cannot use a cystoscope, have never seen the ureter catheterised, are unacquainted with the advances in early diagnosis which modern methods have brought about, yet do not hesitate to perform nephrectomy and other serious operations. Part of this state of things is due to ignorance of the researches of the last ten years, part to distrust of the new and unfamiliar instruments, part to inability to grasp the scientific basis of the work that has been done. But the result is lamentable, and reflects little credit on British surgery.

At several important schools the result of the facts here set forth has been the creation of a special department for genito-urinary surgery, in charge of a member of the staff appointed for the express purpose, and confining himself to this subject—that is to say, not merely one of the general staff devoting spare time to the department. Hitherto, the majority of the British medical schools have not adopted this system; in fact, at most schools there is no special department at all for genito-urinary diseases, which are treated by the general surgeons in the general wards and out-patient rooms. The tendency of the day, however, is towards specialism in this subject; and it is quite possible that in the near future there will be a considerable extension of the special department idea in genito-urinary surgery. At any rate, it can safely be argued that unless the general surgeons as a body begin to take some interest in modern progress as it bears on these diseases, the appointment of specialists at the general hospitals to deal with them is inevitable. We do not mean to imply that all the general surgeons are thus backward; that would be an injustice to many of them who have kept themselves thoroughly up to date. But of too large a proportion of them the charge holds good, and they must not be surprised if, in consequence, they have to surrender some of their hospital beds to a specialist.

### A New Text Book.

Much of the progress in genito-urinary surgery has been the work of foreign surgeons. Of those in Britain who have kept in the forefront of research Mr. Thomson Walker has been one of the most prominent. Besides his own original work he has always been ready to adopt methods evolved by others, to test them fairly, and to advocate them if found satisfactory. The publication of a text-book * by such an admitted authority is therefore a great event, especially since there is none by an English author which covers the whole field. The book is not encyclopædic, for it aims merely at providing the general practitioner and the general surgeon with a foundational account of genito-urinary surgery such as is generally accepted as unquestionably safe and solid. Advanced but improved theories, discarded methods now of merely historical interest, and the newest views not yet definitely proved to be sound are omitted. In other words, the book is not specially adapted for the instruction of specialists.

These aims it fulfils admirably, and we wish every hospital surgeon in Britain could be made to read the book from cover to cover. Lucid in style and excellent in arrangement, there is a practical note and a personal touch about it which make it invaluable for its purpose. For specialists it is scarcely complete enough: to give a couple of instances, there is an excellent method of nephropexy and one of removing a tuberculous kidney which are not mentioned at all. But, broadly speaking, we have nothing but welcome and praise for a book which was badly needed, and will, we hope, do a lot of good. We see no reason why general surgeons should master the technique and the teaching contained in this book; but until they do they have no business to embark upon genito-urinary surgery at all.

* *Genito-Urinary Surgery.* By J. W. Thomson Walker, F.R.C.S. London: Cassell and Co. 1914. Pp. 879. Price 25s. net.

